# Placental Histopathological Changes Associated with *Plasmodium vivax* Infection during Pregnancy

**DOI:** 10.1371/journal.pntd.0002071

**Published:** 2013-02-14

**Authors:** Rodrigo M. Souza, Ricardo Ataíde, Jamille G. Dombrowski, Vanessa Ippólito, Elizabeth H. Aitken, Suiane N. Valle, José M. Álvarez, Sabrina Epiphânio, Claudio R. F. Marinho

**Affiliations:** 1 Departamento de Parasitologia, Universidade de São Paulo, (ICB/USP), São Paulo, São Paulo, Brazil; 2 Campus Floresta, Universidade Federal do Acre, Cruzeiro do Sul, Acre, Brazil; 3 Departamento de Imunologia, Universidade de São Paulo, (ICB/USP), São Paulo, São Paulo, Brazil; 4 Departamento de Ciências Biológicas, Universidade Federal de São Paulo, Diadema, São Paulo, Brazil; Barcelona Centre for International Health Research and Institució Catalana de Recerca i Estudis Avançats, Spain

## Abstract

Histological evidence of *Plasmodium* in the placenta is indicative of placental malaria, a condition associated with severe outcomes for mother and child. Histological lesions found in placentas from *Plasmodium*-exposed women include syncytial knotting, syncytial rupture, thickening of the placental barrier, necrosis of villous tissue and intervillositis. These histological changes have been associated with *P. falciparum* infections, but little is known about the contribution of *P. vivax* to such changes. We conducted a cross-sectional study with pregnant women at delivery and assigned them to three groups according to their *Plasmodium* exposure during pregnancy: no *Plasmodium* exposure (n = 41), *P. vivax* exposure (n = 59) or *P. falciparum* exposure (n = 19). We evaluated their placentas for signs of *Plasmodium* and placental lesions using ten histological parameters: syncytial knotting, syncytial rupture, placental barrier thickness, villi necrosis, intervillous space area, intervillous leucocytes, intervillous mononucleates, intervillous polymorphonucleates, parasitized erythrocytes and hemozoin. Placentas from *P. vivax*-exposed women showed little evidence of *Plasmodium* or hemozoin but still exhibited more lesions than placentas from women not exposed to *Plasmodium*, especially when infections occurred twice or more during pregnancy. In the Brazilian state of Acre, where diagnosis and primary treatment are readily available and placental lesions occur in the absence of detected placental parasites, relying on the presence of *Plasmodium* in the placenta to evaluate *Plasmodium*-induced placental pathology is not feasible. Multivariate logistic analysis revealed that syncytial knotting (odds ratio [OR], 4.21, P = 0.045), placental barrier thickness (OR, 25.59, P = 0.021) and mononuclear cells (OR, 4.02, P = 0.046) were increased in placentas from *P. vivax*-exposed women when compared to women not exposed to *Plasmodium* during pregnancy. A vivax-score was developed using these three parameters (and not evidence of *Plasmodium*) that differentiates between placentas from *P. vivax*-exposed and unexposed women. This score illustrates the importance of adequate management of *P. vivax* malaria during pregnancy.

## Author Summary

Malaria during pregnancy remains a risk for approximately 125 million women each year. Adverse outcomes of malaria during pregnancy include maternal anemia and low infant birth weight. Additionally, the presence of malaria parasites, namely *Plasmodium falciparum*, has been associated with the occurrence of placental lesions. In the Amazonian region of Brazil *Plasmodium vivax* is the primary parasite species. To date, little is known about the capacity of this parasite to induce placental lesions. In this study we have used ten histological parameters to evaluate the effect of exposure to *Plasmodium vivax* during pregnancy on the occurrence of placental lesions when compared to placentas from non-exposed women. Placentas from women exposed to *Plasmodium falciparum* were used as controls. Placentas from *Plasmodium vivax*-exposed placentas did not have strong evidence of placental parasites but had increased syncytial knotting, thickness of the placental barrier and mononuclear cells when compared to non-exposed women. We developed a score based on these three parameters and not on the presence of placental parasites that enables us to visualize the effect that *Plasmodium vivax* has on placentas from women infected during pregnancy.

## Introduction

Approximately 125 million pregnant women worldwide are exposed to the risks of malaria in pregnancy (MiP) each year, resulting in 200,000 infant deaths [1], [2]. In areas in which malaria is endemic, pregnant women are more susceptible to *Plasmodium* infections than their non-pregnant peers. The adverse outcomes of these infections are primarily felt by primigravidae [3], [4], although in areas of low or unstable transmission, women of all gravidities may be equally at risk [4]. Of the five known *Plasmodium* species that infect humans, only *Plasmodium falciparum* and *Plasmodium vivax* have been positively associated with MiP [5]–[7].

Evidence of *Plasmodium* (most commonly mature parasites) or *Plasmodium* products (e.g., hemozoin) in the placenta is considered a defining feature of MiP, often termed placental malaria (PM). This accumulation of parasite material occurs in the intervillous space, a region of low blood flow in the placenta where the maternal blood bathes the syncytiotrophoblast (a cell layer of foetal origin and the site of maternal foetal transfer) [8], [9]. In cases of PM, accumulation of maternal immune cells (predominantly monocytes/macrophages and neutrophils) in the placenta and increased levels of inflammatory cytokines are common [10]–[13]. Placentas affected by PM can also show other signs of injury including syncytial knotting, thickening of the basement membrane of the trophoblast layer and fibrinoid deposits [8], [14]. A particular emphasis has been placed on the presence of *Plasmodium* parasites or hemozoin to assess the pathology of malaria during pregnancy and to identify malaria-associated changes in the placenta.

Although the above information holds true for *P. falciparum* infections, little is known regarding the potential of *P. vivax* to induce PM or even placental pathology. Despite recent evidence that *P. vivax* infections are associated with an increased chances of miscarriage, the occurrence of intrauterine growth restriction [15], [16] and the documented effect on birth weight [5], [7], little information is available regarding the pathological mechanisms that lead to these adverse effects. Few studies have directed their attention to the placental histopathology present in cases of *P. vivax* infection during pregnancy. And, of those that have, none has been conducted in the Amazon region of Brazil [17]–[19], which comprises a unique setting due to the high prominence of *P. vivax* and the ready availability of malaria diagnosis and primary treatment [20], [21]. Recently, *P. vivax* has been shown to adhere to placental cryosections and chondroitin sulphate A [22], [23], stimulating the idea that this parasite has the potential to induce direct placental damage in a manner similar to that of *P. falciparum*. In this study, the placental changes associated with *P. vivax* differed in intensity and distribution from those elicited by *P. falciparum* and occurred in the absence of evidence of placental parasites, suggesting different pathological mechanisms between each species. By developing a histology score based on the most significant placental histology parameters associated with *P. vivax*, we show that women from an endemic region of the Brazilian Amazon, who were diagnosed with *P. vivax* during pregnancy, had higher levels of placental lesions than non-infected pregnant women and that these lesions increase in frequency with increasing exposure to the parasite.

## Materials and Methods

### Ethical Considerations

Ethical clearance was provided by the committees for research of the Fundação Hospitalar do Estado do Acre (n° 333/2008) and the Faculdade de Saúde Pública da Universidade de São Paulo (n° 1871). All participants provided written informed consent or had their legal guardians do so, if they were minors. All pregnant women diagnosed with malaria during pregnancy received adequate treatment according to the recommendations of the Brazilian Health Ministry [24].

### Study Region and Population

The State of Acre, Brazil, has two seasons, a dry season from May to October and a rainy season from November to April. Approximately 70% of the malaria cases observed in the State of Acre are found in the city of Cruzeiro do Sul and the surrounding Alto Juruá river region, where our study was conducted. This region is characterised by a high prevalence of *P. vivax* infections over *P. falciparum* infections [25].

An observational, case-control study was performed at the Maternity Unit of the Hospital da Mulher e da Criança in Cruzeiro do Sul from January to December 2009, in which 1,870 pregnant women were recruited at term The malaria history for each woman during their current pregnancy was recovered from the Brazilian national malaria notification database (SIVEP – MALARIA). A total of 162 (8.7%) women had microscopically-diagnosed *Plasmodium* infections during that pregnancy. Of those cases, 82 placentas were obtained and processed. Additionally, 41 placentas from women without malaria history or a history of fever during the current pregnancy were also processed. Exclusion criteria, based on both self-reported history and medical exams, included infection with HIV or hepatitis, or suffering diabetes and/or pre-eclampsia.

### Sample Collection and Processing

Placental blood (collected after incision of the maternal side of the placenta) was collected, and both thick and thin smears were prepared and stained with Giemsa to assess parasitemia. Placental tissue biopsies (2 cm^3^) were obtained after delivery from an off-centre region of the placenta approximately halfway between the place of insertion of the umbilical cord and the edge of the placenta. Placental samples were stored in 10% neutral buffered formalin at 4°C until they were sent to São Paulo University for processing. After paraffin embedding and sectioning, using standard techniques, 5 µm-thick sections of placental tissue were stained with Haematoxylin-Eosin (H&E), Masson's trichrome stain (MTS) or Giemsa. A Zeiss Axio Imager M2 light microscope equipped with a Zeiss Axio Cam HRc camera was used to capture images of the placentas. Some of the parameters were evaluated and analysed using Image J software (Image J 1.46c Wayne Rasband National Institutes of Health, USA, http://imagej.nih.gov/ij).

### Evaluation of Histopathology

Two individuals, blind to the obstetric and clinical history of the samples, performed all measurements. Cases that proved contradictory between observers were re-evaluated until a consensus was reached. A summary of the parameters evaluated and the evaluation methods used is described in Table 1.

**Table 1:** Stains and scoring system used to quantify malaria-associated placental parameter.

Pathological features		Evaluation methods	Staining
	Syncytial knots	Number of affected villi per 100 villi at 400× magnification	Haematoxylin-Eosin
	Syncytial rupture	Number of affected villi per 100 villi at 400× magnification	Haematoxylin-Eosin
	Placental barrier thickness	Average distance between fetal vessel wall and villus outer membrane as measured by overlaying horizontal lines 5 µm apart on 20 random terminal villi at 1000×	Masson's Trichrome Stain
	Fibrinoid Necrosis	Number of intersection points on a random grid that touched areas of necrosis per total points of a square grid 4,862.43 µm^2^ of area point at 100× magnification	Hematoxylin-Eosin
	Intervillous space area	Number of intersection points on a random grid that touched areas of intervillous space per total points of a square grid 4,862.43 µm^2^ of area point at 100× magnification	Hematoxylin-Eosin
	Intervillous leucocytes	Percent of total leucocytes in 500 intervillous cells at 400× magnification	Hematoxylin-Eosin
	Intervillous mononucleates	Percent of mononuclear cells in 500 intervillous cells at 400× magnification	Hematoxylin-Eosin
	Intervillous polymorphonucleates	Percent of polymorphonucleated cells in 500 intervillous cells at 400× magnification	Hematoxylin-Eosin
**Malaria-associated features**			
	Parasitised erythrocytes	Number of fields with parasite in 100 fields at 1000× magnification	Giemsa
	Hemozoin	Ten fields at 200× magnification of picric acid-treated sections were screened with polarised light for the presence of hemozoin in the intervillous space (free or within cells) and in the tissue.	Hematoxylin-Eosin

### Placental Lesion Scoring

The placental lesion score was calculated after identifying the placental parameters that were associated with exposure to *P. vivax* during pregnancy in comparison with non-exposed women. More details about the score are described in the results section.

### Definitions

Anaemia was defined as a haemoglobin result of <11 g/dL. Low birth weight was defined as an infant weight of <2,500 g.

### Statistical Analyses

The data were analysed using Stata 9.2 software (StataCorp, College Station, TX, 2005) and GraphPad Prism (GraphPad Prism version 5.00 for Windows, GraphPad Software, San Diego, California, USA, www.graphpad.com). The variables with normal distributions were analysed based on means and standard deviations, and the variables that were non-normally distributed were analysed based on medians and quartiles. All of the placental parameters evaluated were Ln-transformed before statistical analysis. One-way ANOVA tests with Bonferroni correction were used to determine the differences between groups. Student's t-tests or Mann-Whitney U-tests were used when appropriate. Categorical data were analysed using Chi-Square tests. Cuzick's test was applied across ordered groups in order to evaluate trends. Multivariate analyses was performed using multiple logistic regression models.

## Results

### Study Samples and Cohort

A total of 123 placentas from singleton deliveries were available for study. Four women were excluded from the study for presenting with more than one parasite species, diagnosed by microscopy, during pregnancy; consequently, 119 placentas were studied.

Forty-one (35%) of the placentas were obtained from women without documented evidence of any *Plasmodium* infection during pregnancy, 59 (49%) were taken from women with microscopically documented *P. vivax* infection during pregnancy and 19 (16%) were obtained from women with microscopically documented *P. falciparum* during pregnancy. No differences in maternal age, gravidity, or duration of gestation were observed between the three groups of women (Table 2). The mean hematocrit values were significantly lower in the “falciparum” group (mean, SD: 31.26, 4.09) when compared to those from both the “no plasmodium” (t-test, P = 0.015) and “vivax” (t-test, P = 0.029) groups (Table 2). Higher frequencies of anemia were found in both groups of infected women when compared to the “no plasmodium” group, although there was little evidence that this was a true difference (Table 2). Infant birth weight and the proportion of low birth weight babies also did not differ significantly between groups (Table 2). Contrary to the women in the “no plasmodium” group, in which only 61% reported a previous malaria infection during their lifetime, all of the women in the exposed groups had previously experienced malaria (χ^2^ test: P<0.001).

**Table 2:** Characteristics of the women who participated in the study according to infection status†.

		No Malaria	*P. vivax*	*P. falciparum*
**Demographics**				
	Age, years (mean (SD) [n])	23 (6) [41]	24 (6) [59]	23 (6) [19]
	Gestational age, weeks (median (IQ) [n])	39 (38–40) [40]	39 (38–40) [59]	39 (38–40) [19]
	Gravidity, (median (IQ) [n])	2 (1–4) [40]	2 (1–4) [58]	3 (1–6) [19]
	Primigravidae, %	44	31	26
**Clinical outcomes**				
	Maternal Hb, g/dL (mean, SD) [n])	11.37 (1.40) [33]	11.28 (1.77) [45]	10.35 (1.46) [15]
	Maternal hematocrit, % (mean (SD) [n])	34.17 (4.20) [41]	34.13 (5.10) [59]	31.26 (4.09) [19]◊ •
	Maternal anemia, %	36	42	53
	Birth weight, g (mean (SD) [n])	3192.20 (504.40) [41]	3107.29 (564.12) [59]	3131.67 (507.39) [18]
	Low birth weight, %	10	12	6
**Malaria history**				
	Had previous infection in life, % [n]	61 [41]§	100 [59]	100 [19]
	Ep. in current pregnancy, (median (min-max) [n])	N/A	1 (1–5) [59]	1 (1–3) [19]
	2+ infections during pregnancy (n/total)	N/A	20/59	6/19
	Peripheral parasitemia at term, (n/total)	N/A	3/59	4/19
	Month of infection, (mean (SD), [n])	N/A	5 (3) [58]	6 (3) [17]
	Trimester of infection, (% 1^st^/2^nd^/3^rd^)	N/A	34/33/33	36/10/54

† Women were grouped according to their malaria diagnoses during pregnancy, based on microscopy data. (SD) standard deviation. (IQ) 25^th^ and 75^th^ percentile. (n) number of women with data recorded. (%) percentage of total women in each group. (Hb) hemoglobin. (Ep.) number of episodes. (N/A) not applicable. Anemia: Hb<11 g/dL. Low birth weight: birth weight<2,500 g. 2+ infections during pregnancy: women with two or more infections detected over the duration of the pregnancy. Month of infection: Gestation month of first diagnosed infection. Trimester of infection: percentage of infections that were detected per trimester of gestation.

◊ Student's t-test: *P. falciparum* vs. no malaria, P = 0.015.

• Student's t-test: *P. falciparum* vs. *P. vivax*, P = 0.029.

§ χ^2^ test: no malaria vs. *P.* vivax or *P.* falciparum, P<<0.001.

When women in the “no plasmodium” and “vivax” groups were sub-divided into those with one pregnancy (primigravidae) and those with two or more pregnancies (multigravidae) the only significant difference was of birth weight of the primigravidae, with the mean birth weight of infants in the “no plasmodium” group 350 g higher than that of the “vivax” group (student's t-test, P = 0.03)

### Histology

The evaluated parameters were normalised prior to analysis using Ln transformation.

#### Placental changes

**Figure 1:**
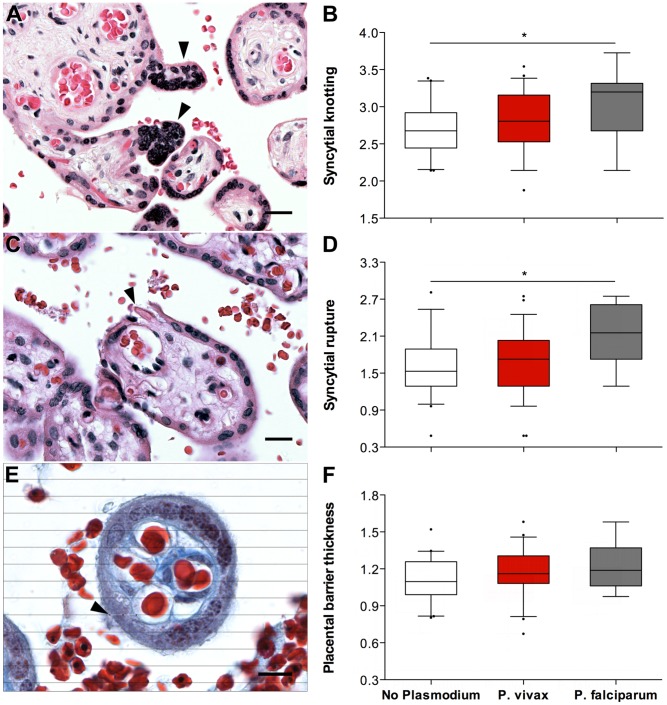
The syncytial parameters evaluated by *Plasmodium* species during infection. Syncytial knotting (A and B) and syncytial rupture (C and D) were evaluated on H&E-stained slides at 100× magnification. Placental barrier thickness (E and F) was evaluated on Masson's trichrome-stained slides at 1000× magnification after overlaying horizontal lines with 5 µm of interspace (see Methods and Table 1). For all parameters, placentas from the “no plasmodium” group (n = 41; white boxes) had the lowest values, followed by placentas from the “P. vivax” (n = 59; red boxes) and “P. falciparum” (n = 19; grey boxes) groups. Graphs (B, D and F) represent the transformed data. * ANOVA test, P-value≤0,006. The boxes represent the mean and standard deviation values. The whiskers represent the 5^th^ and 95^th^ percentiles. Photographs were taken using a Zeiss Axio Imager M2 light microscope equipped with a Zeiss Axio Cam HRc. The grid overlays and counts were conducted using Image J. Arrow heads on A, C and D point to syncytial knots, syncytial rupture and an example of a thickness measurement, respectively.

**Figure 2:**
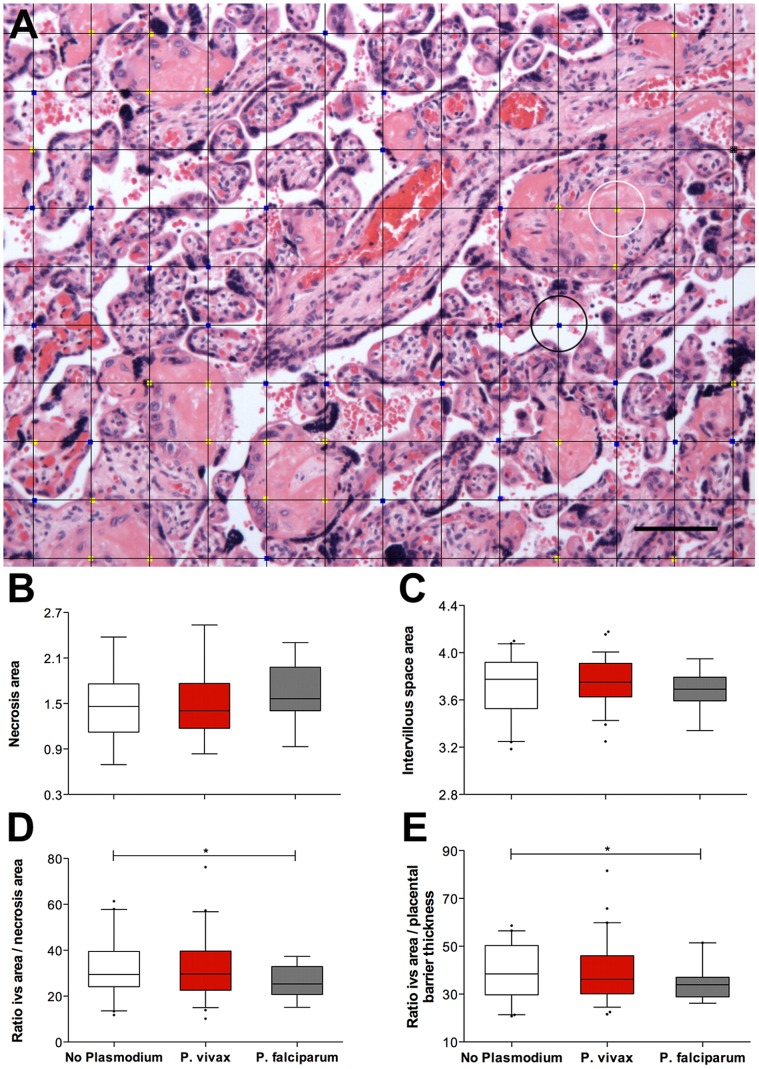
The placental parameters evaluated by *Plasmodium* species during infection. For all placentas, areas of necrosis (B) and intervillous space (C) were measured by overlaying a square grid (A) and counting the number of intersecting points that touched necrotic areas (yellow dots; the white circle indicates an example) or intervillous space areas (blue dots; the black circle indicates an example). The ratios of intervillous space area per necrosis (D) and intervillous space area per placental barrier thickness (E) were calculated. The placentas in the “no plasmodium” group (n = 41; white boxes) appear to have similar necrotic areas and more intervillous space than the placentas in the “P. vivax” group (n = 59; red boxes). The placentas in the “P. falciparum” group (n = 19; grey boxes) exhibited more necrotic areas and less intervillous space. Graphs (B, C, D and E) represent the transformed data. The boxes represent the mean and standard deviation values. The whiskers represent the 5^th^ and 95^th^ percentiles. The photograph was taken using a Zeiss Axio Imager M2 light microscope equipped with a Zeiss Axio Cam HRc. Grid overlays and counts were performed using Image J.

When univariate analyses of each parameter against the species of *Plasmodium* infection diagnosed during pregnancy were performed (Table 3, and Figs. 1 and 2), a significant increase in the percentage of syncytial knots (ANOVA, P = 0.006) (Figs. 1A and 1B) was observed in the placentas from the *P. falciparum*-exposed women. The values of syncytial knotting were increased in women in the “falciparum” group versus women in the “no-plasmodium” group, while women in the “vivax” group exhibited intermediate syncytial knotting values. This same pattern was also observed for the presence of syncytial rupture (ANOVA, P = 0.003) (Figs. 1C and 1D) and the thickness of the placental barrier (ANOVA, P = 0.08) (Figs. 1E and 1F), although no statistical significance was obtained with this last parameter. Area of necrosis (ANOVA, P = 0.227) (Figs. 2A and 2B) did not differ between groups. Area of intervillous space appeared to be smaller in the *Plasmodium*-exposed groups, without reaching significance, and again, women in the “vivax” group had intermediate values (ANOVA, P = 0.357) (Figs. 2A and 2C). This pattern was also found when analyses of two ratios by *Plasmodium* infection were performed (reflecting a possible decrease of exchange areas between mother and fetus): intervillous space/necrosis (ANOVA, P = 0.132) (Fig. 2D) and intervillous space/placental barrier thickness (ANOVA, P = 0.112) (Fig. 2E).

**Table 3:** Results and univariate analysis of the placental parameters, evaluated by histology, according to the species of *Plasmodium* infection during pregnancy†.

		No Malaria	*P. vivax*	*P. falciparum*
		*n* = 41	*n = 59*	*n = 19*
**Placental changes [median (IQ)]**				
	Syncytial knotting, %	10 (7–14)	12 (8–19)	20 (10–23)*
	Syncytial rupture, %	5 (4–7)	6 (4–8)	9 (6–14)* §
	Placental barrier thickness, µm	3.56 (3.28–4.05)	3.76 (3.51–4.26)	3.85 (3.46–4.50)
	Necrosis area, %	2.31 (1.23–3.69)	2.08 (1.23–3.85)	2.77 (2.08–5.23)
	Intervillous space (IVS) area, %	43.62 (35.31–49.85)	42.54 (37.54–49.92)	40.08 (36.31–44.38)
	Ratio IVS/necrosis	19.62 (11.32–33.52)	18.07 (10.74–34.07)	15.07 (7.35–23.24)
	Ratio IVS/Placental barrier thickness	38.40 (30.02–49.21)	36.18 (30.12–46.03)	33.87 (28.82–37.03)
**Immune cells [median (IQ)]**				
	Total leucocytes, %	2.6 (1.4–3.2)	2.8 (1.8–4.6)	2.8 (2.0–5.0)
	Mononucleates, %	1.0 (0.6–1.4)	1.2 (0.6–2.0)	1.4 (1.0–2.7)°
	Polymorphonucleates, %	1.2 (0.6–2.2)	1.4 (0.6–2.8)	1.2 (0.4–1.8)
**Malaria**				
	Parasites, % fields (median (IQ) [n])	N/A	1 (1–1) [1]	75 (2–94) [6]#
	Presence of hemozoin, n	N/A	4	11#

† Women were grouped according to their malaria diagnoses during pregnancy, based on microscopy data. (IQ) 25^th^ and 75^th^ percentile. (n) number of women. (%) percentage (N/A) not applicable. For differences between groups all continuous variables were Ln-transformed and one-way ANOVA tests with Bonferroni post-tests were performed. Chi^2^ tests were used to evaluate differences in categorical variables between groups. Refer to the “methods section” for a full description of how each parameter was measured.

* *P. falciparum* vs. No Malaria, P = 0.004.

§ *P. falciparum* vs. *P. vivax*, P = 0.005.

° *P. falciparum* vs. No Malaria, P = 0.048.

# χ^2^ test: *P.* vivax or *P.* falciparum, P<0.001.

No significant differences were observed between the “no plasmodium” and “vivax” groups when they were sub-divided according to parity (data not shown).

#### Immune cells

**Figure 3:**
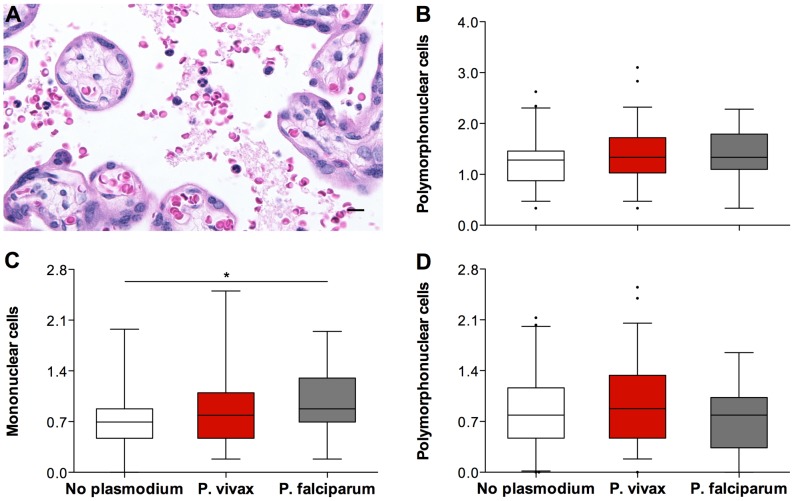
The immune-cell parameters evaluated by *Plasmodium* species during infection. The percentage of immune cells present in the intervillous space of the placentas evaluated (A) was calculated after counting a total of 500 intervillous space cells. Total leucocytes percentage (B), mononuclear cells percentage (C) and polymorphonuclear cells percentage (D) were plotted against *Plasmodium* exposure during pregnancy, assessed by microscopy. The placentas from the “no plasmodium” group (n = 41; white boxes) appear to have less immune cells present in the intervillous space than the placentas from the “P. vivax” group (n = 59; red boxes) and the placentas from the “P. falciparum” group (n = 19; grey boxes). * ANOVA test, P-value = 0,039. Graphs (B, C, and D) represent the transformed data. The boxes represent the mean and standard deviation values. The whiskers represent the 5^th^ and 95^th^ percentiles. The photograph was taken using a Zeiss Axio Imager M2 light microscope equipped with a Zeiss Axio Cam HRc. Grid overlays and counts were performed using Image J.

Univariate analyses of the number of cells found in the intervillous spaces of the placental samples (Fig. 3) revealed no differences between total leucocytes (ANOVA, P = 0.406) (Fig. 3B) and polymorphonucleates (ANOVA, P = 0.295) (Fig. 3D) between the groups. Numbers of mononuclear cells were increased in the groups exposed to *Plasmodium* during pregnancy (Fig. 3C). Once again, the “vivax” group had intermediate values between the “no plasmodium” and the “falciparum” group which exhibited the highest values (ANOVA, P = 0.039). No significant differences were observed between the “no plasmodium” and “vivax” groups when these where sub-divided according to parity.

#### Malaria-associated features

Both *Plasmodium* parasites and malarial pigment (hemozoin) were only observed in the placental samples from women diagnosed with malaria during pregnancy. Only one of the 59 “vivax” group samples was found to have parasites in the intervillous space and whilst six of the 19 “falciparum” group samples had parasites present (χ^2^ test, P<0.001). These results were reflected when assessing the presence of hemozoin; four of the 59 “vivax” group placentas exhibited evidence of hemozoin when compared with 11 of the 19 “falciparum” group placentas (χ^2^ test, P<0.001). In both the “vivax” and the “falciparum” groups approximately one-third of the women experienced more than one infection during pregnancy. All *P. vivax* infections were equally distributed along the trimesters of gestation while *P. falciparum* infections occurred primarily in the first and third trimesters.

Women were divided according to the number of *P. vivax* (and not *P. falciparum*) infections experienced during pregnancy and the following group order was established: “no infection”, “1 infection” and “2+ infections”. All histological parameters were evaluated between these groups and Cuzick's trend test across ordered groups was performed to assess presence of a trend with any of the parameters according to the number of *P. vivax* infections (Table 4). Both syncytial knots (z = 2.35; P = 0.019) and placental barrier thickness (z = 2.63; P = 0.008) were elevated with increased frequency of *P. vivax* infections.

**Table 4:** Cuzick's trend test analysis of placental changes across ordered groups by number of *P. vivax* infections during pregnancy†.

	Trend across infection groups (z)	*P value*
	(0 →1 →2+)^a^	
**Syncytial knotting**	*2.35*	*0.019*
**Syncytial rupture**	1.59	0.112
**Placental barrier thickness**	*2.63*	*0.008*
**Necrosis area**	0.53	0.597
**Intervillous space area**	0.22	0.826
**Mononuclear cells**	1.67	0.096
**Polymorphonucleates**	1.43	0.154

† Women were divided according to the number of *P. vivax* infections that were diagnosed microscopically during pregnancy.

a Cuzick's trend test was performed across the ordered groups: no *P. vivax* infection (0), one *P. vivax* infection (1) and two or more *P. vivax* infections (2+). (z) Measure and direction of tendency.

### Placental Score

Multivariate logistic regression analyses were conducted to identify the placental histology parameters that were associated with *P. vivax* exposure during pregnancy when compared to no *Plasmodium* exposure. This was performed first in a random selection of women from the “no plasmodium” and the “vivax” groups, which were used to develop a placental score, which was subsequently applied to the entire cohort to confirm the results obtained (Table 5). Seven parameters were evaluated against each other. Of these, syncytial knotting (odds ratio [OR], 7.48 [95% confidence interval {CI}, (0.86–64.91)], P = 0.068), placental barrier thickness (OR, 25.28 [95% CI (0.59–1083.38)], P = 0.092) and presence of mononuclear cells (OR, 6.08 [95% CI (0.91–40.97)], P = 0.063) showed the strongest evidence of being different between the placentas from *P. vivax*-exposed and non-exposed women. Thus, a formula was developed that aimed to enhance these parameters and weight them differently according to their relative significance in the model (i.e., the odds ratio). The use of a ‘base ten’ assured us that small changes would be highly reflected in the model, thereby enhancing the existing differences between the “no plasmodium” and “vivax” groups. A division by one-thousand, in the formula, allowed the score output to have manageable values. The following formula was developed:
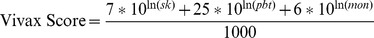
In which, *sk* = syncytial knotting, *pbt* = placental barrier thickness and *mon* = mononuclear cells.

**Table 5:** Multivariate analysis of the association between placental histological parameters and malaria during pregnancy.

	*Sample group* a		*Full dataset* b	
*“P. vivax” vs. “no malaria”*	*Odds Ratio (95%CI)*	*P value*	*Odds Ratio (95%CI)*	*P value*
	*n = 50*		*n = 100*	
**Syncytial knotting**	*7.48 (0.86–64.91)*	*0.068*	*4.21 (1.03–17.19)*	*0.045*
**Syncytial rupture**	*0.52 (0.10–2.63)*	*0.428*	*NS*	
**Placental barrier thickness**	*25.28 (0.59–1083.38)*	*0.092*	*25.59 (1.639–433.85)*	*0.021*
**Necrosis area**	*1.42 (0.18–10.97)*	*0.738*	*NS*	
**Intervillous space area**	*1.03 (0.95–1.11)*	*0.522*	*NS*	
**Mononuclear cells**	*6.08 (0.91–40.97)*	*0.063*	*4.02 (1.02–15.79)*	*0.046*
**Polymorphonucleates**	*0. 60 (1.60–2.30)*	*0.461*	*NS*	

a Women were randomly chosen from the “No-plasmodium” (n = 21) and “Vivax” (n = 30) groups in order to find the best candidates for building the vivax-score.

b In order to confirm the score, all the women from the “No-plasmodium” (n = 41) and “Vivax” (n = 59) were included in the analysis. Multivariate logistic regression was performed using Ln-transformed variables. (CI) Confidence Interval. P values are adjusted for the multivariate analysis.

**Figure 4:**
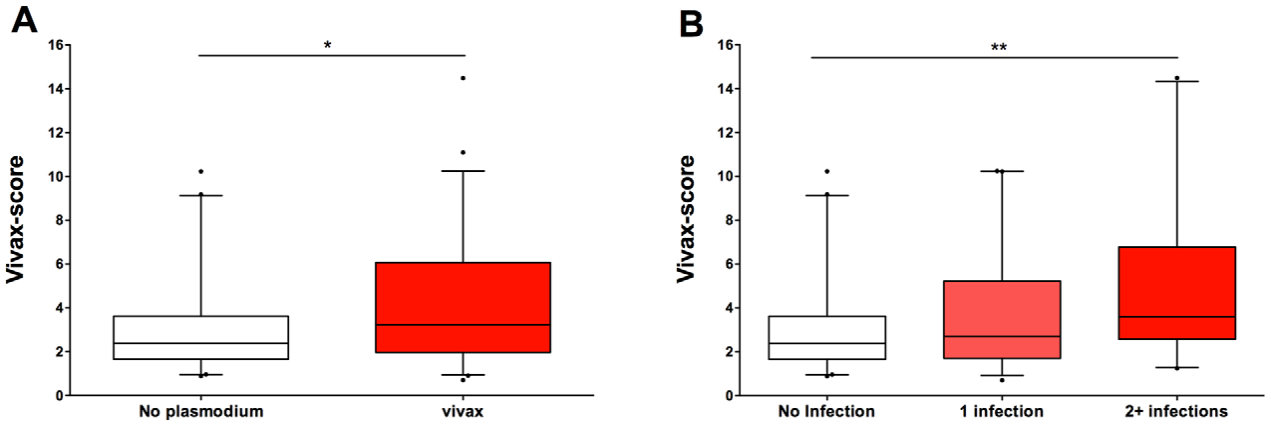
The placental score differentiates the women who were exposed to *P. vivax* during pregnancy. A score (termed the ‘vivax-score’) was developed and applied to all of the placental samples in this study (see main text for details). (A) The placental samples from the “no Plasmodium” group (n = 41, white box) revealed a significantly lower score than the placentas from the “P. vivax” group (n = 59, red box) (* Mann-Whitney, P = 0.027). (B) The vivax-score increased significantly (** Cuzick's trend test: z = 2.76, P = 0.006) with increased exposure to *P. vivax* during pregnancy. “No infection”, n = 41; “1 infection”, n = 39; “2+ infections”, n = 20).

This formula was applied to all of the samples in the study (Fig. 4A). Placentas belonging to the “vivax” group exhibited higher scores than those from the “no plasmodium” group (Mann-Whitney, P = 0.027). Also, applying the score to the different groups, according to the number of *P. vivax* infections experienced during pregnancy, showed an increase in score values with increasing numbers of *P. vivax* infections (Cuzick's trend-test: z = 2.76; P = 0.006) (Fig. 4B)

## Discussion

Our results show that by applying an injury score based on parameters that do not consider *Plasmodium* presence (syncytial knotting, placental barrier thickness and presence of mononuclear cells), we can detect the influence of *P. vivax* on placental pathology.

Correctly evaluating the impact of a malarial infection on the placenta is significant when using endpoint histology as a tool during clinical trials, drug trials, and vaccine trials. In areas such as Acre state in the Brazilian Amazon region, where *P. vivax* is the primary parasite species and where access to malaria diagnosis is readily available and primary treatment is quickly applied, the current tools for the diagnosis of placental malaria and histological analyses of placental changes associated with malaria are not helpful [8], [9], [12], [18].

Our study was based on the collection of placentas at delivery and the application of a questionnaire to retrieve the clinical history of the women. Women with microscopically diagnosed *Plasmodium* infections, during pregnancy, were referred to us by the malaria epidemiological surveillance team (agentes de endemias) of Cruzeiro do Sul, Acre, Brazil. The women were assigned to three distinct groups based on the species of *Plasmodium* identified by microscopy during pregnancy. This selection may have overlooked women with asymptomatic infections. As yet the incidence of these infections in pregnancy in this population has not been identified and, as such, for the purpose of this study these were not considered to be significant confounding factor. Ideally however future studies should confirm women were free of parasitemia throughout pregnancy via PCR and/or microscopy. As shown for other areas of Brazil for both pregnant and non-pregnant individuals [20], [26], *P. vivax* was the most prevalent species identified. No differences were observed between groups with respect to the demographics of the women enrolled in the study suggesting that there were no biases between groups and that the demographic parameters analysed were not confounding factors in the study (Table 2). Of the recorded clinical outcomes, only maternal hematocrit was significantly different between the “falciparum” group and the others, possibly reflecting the greater severity of *P. falciparum* infection. The self-reported malaria history of the women revealed that a significant proportion of the women (39%) in the “no plasmodium” group had never contracted malaria. All of the women diagnosed with *Plasmodium* infections during pregnancy had previously experienced an episode of malaria. Although all of the women were from Cruzeiro do Sul, different pockets of transmission may have contributed to this finding. Notably, and although not part of the original goal of this study, an analysis of the infant birth weight according to the parity of the women revealed that primigravidae who were infected with *P. vivax* during pregnancy had lower-weight babies (350 g, Student's t-test, P = 0.03) than did primigravidae from the “no plasmodium” group. The biological significance of this finding, although similar to previous results [5], [7], [27], could not be determined in this study since birth weight is a multidimensional outcome that depends not only on exposure and immunity to malaria [28], [29], but also on anthropometric features of both the parents and the infant [30], [31].

Initially, the histopathological analysis of the placentas performed was to be based on published methods and scores for the detection and classification of lesions associated with PM [8], [9], [12], [18]. This was not helpful since neither intervillositis, placental parasites nor hemozoin deposition were frequently observed. Nevertheless, women infected with *P. vivax* during pregnancy consistently exhibited higher levels of placental lesions than women in the “no plasmodium” group (Table 3), which we took to be an indication that *P. vivax*-associated placental changes were occurring, in the absence of classical PM. This notion was further supported by our observation that certain histological parameters were increased in women who had experienced two or more *P. vivax* infections during pregnancy when compared to women who had experienced a single episode of *P. vivax* or women who were not diagnosed with *Plasmodium sp.* during pregnancy (Table 4). Next, we turned our attention to the parameters that might be associated with *P. vivax* infections in this region, when compared to non-infected women, without depending on the presence of malaria parasites (or products) in the placenta.

Although others have addressed the need for novel histopathological classifications for PM that do not include the presence of parasites [18], these methods rely on both hemozoin and inflammation. Our results showed that both of these features were largely absent from our samples (Table 3). The existence of a well-established malaria diagnosis and primary treatment system in the study area (reviewed in [21]) may have contributed to the low numbers of inflammatory cells observed in the placentas from both the “vivax” and “falciparum” groups. Still, a significantly higher number of mononuclear cells were present in placentas from *P. falciparum-*exposed women when compared to placentas from unexposed women. This result supports other observations where local inflammatory responses were associated with *P. falciparum* infections during pregnancy [12], [32]. This association was not seen with the “vivax” group. Taken together with the higher proportion of parasites and/or haemozoin present in *P. falciparum* placentas (Table 3), this suggests that *P. vivax* has a reduced occupancy time in the placenta compared to *P. falciparum*. Recent reports which show that *P. vivax* is able to adhere to chondroitin sulphate A [22] and placental cryosections [23] need to be complemented with studies looking at the adhesive properties of placental *P. vivax* to fully resolve this issue.

The consistent observation that the “vivax” group presented with higher severity for the majority of the parameters measured, than the “no plasmodium” group (despite a lack of statistical significance) led us to consider the hypothesis that a combination of non-*Plasmodium*-related factors could be used to identify placentas affected by *P. vivax*. By conducting multivariate logistic regression analyses, first on a sub-group of samples and then confirmed on the entire sample cohort (Table 5), we were able to identify the parameters that, when taken together, varied between the placentas from *P. vivax*-exposed women and those from unexposed women. This analysis revealed three parameters that exhibited an increased probability (compared to the “no plasmodium” group) of occurring in the “vivax” group: syncytial knotting, placental barrier thickness and presence of mononuclear cells. The vivax-score formula developed, included not only these parameters, but also their relative significance, reflected by the use of the odds ratio obtained for each parameter. When this formula, which was obtained from a random sample of the placentas available, was applied to all placental samples in the study, placentas from the “vivax” group exhibited significantly higher vivax scores than those from the “no plasmodium” group (Mann-Whitney, P = 0.027). The fact that three parameters which do not include the direct or indirect evidence of a *Plasmodium* infection are able to differentiate placentas that have been exposed to *P. vivax* during pregnancy is in itself of extreme significance in a setting where evidence of parasites in the placenta is scarce.

The question now is ‘why these three parameters?’ Syncytial knotting is highly associated with hypoxia and oxidative stress [33], [34] and has been repeatedly observed in placentas from *P. falciparum*-exposed women [8], [35], [36] but has not been associated with the presence of *P. vivax* [17]. Only circumstantial evidence exists for *P. vivax*-induced hypoxia [37], and no known pathway for it exists [38], [39], although a systemic cytokine storm could be the answer [40]. Additionally, studies examining malaria-induced hypoxia during pregnancy have not shown evidence of its existence [41], [42]. The lack of evidence for malaria induced hypoxia represents a puzzle that must be solved because the existence of increased syncytial knotting in placentas exposed to *Plasmodium* appears to suggest that hypoxic conditions do in fact occur. Increased placental barrier thickness, a feature associated with reduced transport of nutrients and oxygen to the foetus [43], has previously been observed in studies of the impact of *P. falciparum* during pregnancy [35], [44], [45] when measured as the thickness of the trophoblast basement membrane and not as the placental barrier as a whole. All the above-cited results which reported increased thickness were obtained from placentas that showed evidence of parasite infection and associated inflammation. Our study suggests that syncytial knotting and placental barrier thickness (which are associated with conditions of hypoxia and decreased nutrient transport) are altered in placentas without clear evidence of either *Plasmodium* infection or inflammation in women exposed to *P. vivax* during pregnancy. The increased presence of mononuclear cells in the *P. vivax*-exposed placentas may represent either a general or a local inflammatory milieu. It is difficult, in this study, to ascertain which of these is correct. It is very well established that *P. falciparum* infections are usually accompanied by an accumulation of mononuclear cells in the placenta, primarily composed of monocytes, as well as an inflammatory environment that contributes to the detrimental effects of this infection [8], [9], [12], [13], [32], [46], [47]. It is worth keeping in mind that what we observed in this study was a slight increase in the number of mononuclear cells and a probability that these are associated with *P. vivax* and not an inflammatory environment or any evidence of intervillositis.

The low number of *P. falciparum-*infected women did not allow us to explore the placental pathological features of this infection, but did provide indications that the two parasites have different intensities, if not mechanisms, of pathogenesis during pregnancy. The small number of women in the “falciparum” group also prevented the development of a falciparum score.

Determining the parameters that allow the distinction between placentas exposed to either *P. vivax* or *P. falciparum* as well as the differentiation between the mechanisms of action of these two parasite species will be very important. Additionally, the vivax-score we developed may become useful when using end-point histology to assess the effectiveness of drug-treatments, vaccine trials and other interventions. This study represents a first step to understanding if and how *P. vivax* is able to induce placental pathology even in the absence of parasite or hemozoin in the placenta at delivery.
