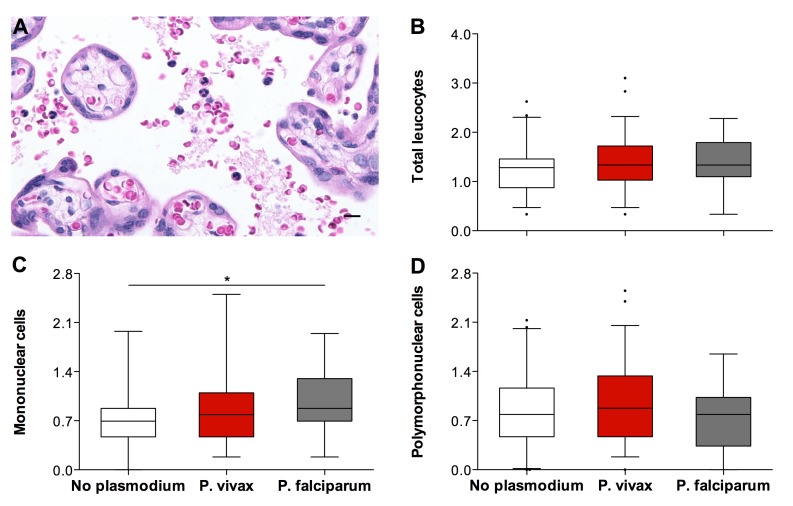# Correction: Placental Histopathological Changes Associated with *Plasmodium vivax* Infection during Pregnancy

**DOI:** 10.1371/annotation/28901e80-13ad-4cae-99e8-8d54625743b6

**Published:** 2013-04-02

**Authors:** Rodrigo M. Souza, Ricardo Ataíde, Jamille G. Dombrowski, Vanessa Ippólito, Elizabeth H. Aitken, Suiane N. Valle, José M. Álvarez, Sabrina Epiphânio, Claudio R. F. Marinho

Panel B of Figure 3 is incorrectly labeled. It should read "Total leucocytes." Additionally, there is a misspelling in the name of author 8. Her name should be spelled "Sabrina Epiphanio."

The corrected figure image can be found here: 

**Figure:**